# Modulation of Ca_V_1.3b L-type calcium channels by M_1_ muscarinic receptors varies with Ca_V_β subunit expression

**DOI:** 10.1186/s13104-018-3783-x

**Published:** 2018-09-27

**Authors:** Mandy L. Roberts-Crowley, Ann R. Rittenhouse

**Affiliations:** 1 Department of Physiology, Program in Neuroscience, University of Massachusetts Medical School, 55 Lake Ave North, Worcester, MA 01655 USA; 2 Department of Microbiology and Physiological Systems, Program in Neuroscience, University of Massachusetts Medical School, 368 Plantation Street, Worcester, MA 01605 USA

**Keywords:** Acetylcholine, Ca_V_β subunit, Dopamine, L-type calcium current

## Abstract

**Objectives:**

We examined whether two G protein-coupled receptors (GPCRs), muscarinic M_1_ receptors (M_1_Rs) and dopaminergic D_2_ receptors (D_2_Rs), utilize endogenously released fatty acid to inhibit L-type Ca^2+^ channels, Ca_V_1.3. HEK-293 cells, stably transfected with M_1_Rs, were used to transiently transfect D_2_Rs and Ca_V_1.3b with different Ca_V_β-subunits, allowing for whole-cell current measurement from a pure channel population.

**Results:**

M_1_R activation with Oxotremorine-M inhibited currents from Ca_V_1.3b coexpressed with α_2_δ-1 and a β_1b_, β_2a_, β_3_, or β_4_-subunit. Surprisingly, the magnitude of inhibition was less with β_2a_ than with other Ca_V_β-subunits. Normalizing currents revealed kinetic changes after modulation with β_1b_, β_3_, or β_4_, but not β_2a_-containing channels. We then examined if D_2_Rs modulate Ca_V_1.3b when expressed with different Ca_V_β-subunits. Stimulation with quinpirole produced little inhibition or kinetic changes for Ca_V_1.3b coexpressed with β_2a_ or β_3_. However, quinpirole inhibited N-type Ca^2+^ currents in a concentration-dependent manner, indicating functional expression of D_2_Rs. N-current inhibition by quinpirole was voltage-dependent and independent of phospholipase A_2_ (PLA_2_), whereas a PLA_2_ antagonist abolished M_1_R-mediated N-current inhibition. These findings highlight the specific regulation of Ca^2+^ channels by different GPCRs. Moreover, tissue-specific and/or cellular localization of Ca_V_1.3b with different Ca_V_β-subunits could fine tune the response of Ca^2+^ influx following GPCR activation.

**Electronic supplementary material:**

The online version of this article (10.1186/s13104-018-3783-x) contains supplementary material, which is available to authorized users.

## Introduction

Voltage-gated Ca^2+^ channels (VGCCs) control membrane excitability, gene expression, and neurotransmitter release [1]. Alterations in these cellular functions occur when GPCR-activated signal transduction cascades modulate VGCCs. In medium spiny neurons (MSNs) of the striatum, GPCRs, including M_1_Rs and D_2_Rs, inhibit VGCC activity [2, 3]. These GPCRs specifically inhibit Ca_V_1.3 L-current, decreasing the output of MSNs [3, 4] and may have functional consequences for motor control [5, 6].

Although present in MSNs, M_1_R signaling has been characterized most thoroughly in superior cervical ganglion (SCG) neurons. M_1_Rs couple to Gα_q_ and phospholipase C (PLC) to inhibit native L- and N-VGCC currents [7–9]. This signal transduction cascade, referred to as the slow or diffusible second messenger pathway, is characterized as pertussis toxin (PTX)-insensitive, voltage-independent, and requiring intracellular Ca^2+^ to function [10]. Our laboratory has identified arachidonic acid (AA) as a critical effector in the slow pathway [9]. Exogenously applied AA inhibits L-current [11–13], which in SCG neurons most likely arises from Ca_V_1.3 [14]. Moreover, Ca^2+^-dependent cytosolic phospholipase A_2_ (cPLA_2_) appears critical for release of AA from phospholipids following M_1_R activation; loss of cPLA_2_ activity by pharmacological antagonists or gene knockout ablates L-current inhibition [15, 16].

Additionally, D_2_Rs inhibit L-current via a diffusible second messenger pathway involving phospholipase C (PLC), InsP_3_, and calcineurin in MSNs [3]. While both GPCRs signal through PLC, they share another commonality: their activation releases AA from striatal neurons [17, 18] and transfected cell lines [19, 20]. Therefore, D_2_Rs may also inhibit L- (Ca_V_1.3) and N-(Ca_V_2.2) currents via a pathway utilizing cPLA_2_ to release AA. In the present study, we tested whether the M_1_R and D_2_R pathways converge to modulate recombinant L-VGCC activity.

## Main text

### Materials and methods

#### Cell culture

Human embryonic kidney cells, stably transfected with the M1 muscarinic receptor (HEK-M1) [a generous gift from Emily Liman, University of Southern California, originally transfected by [21] ] were propagated at 37 °C with 5% CO_2_ in Dulbecco’s MEM (DMEM)/F12 supplemented with 10% FBS, 1% G418, 0.1% gentamicin, and 1% HT supplement (Gibco Life Technologies). Cells were passaged when 80% confluent.

#### Transfection

HEK-M1 cells, grown in 12-well plates (~ 60–80% confluent), were transfected with a 1:1:1 molar ratio of Ca_V_1.3b or Ca_V_2.2, α_2_δ-1 and different Ca_V_βs [22], using Lipofectamine PLUS (Invitrogen) according to the manufacturer’s instructions. Cells were co-transfected with green fluorescent protein (GFP) to identify transfected cells. Constructs for Ca_V_1.3b (+exon11, Δexon32, +exon42a; GenBank accession #AF370009), Ca_V_2.2 (^a^10, Δexon18a, Δexon24a, +exon31a, +exon37b, +exon46; #AF055477), Ca_V_β_3_ (#M88751) and α_2_δ-1 (#AF286488) were provided by Diane Lipscombe (Brown University). Ca_V_β_1b_ (#X61394), Ca_V_β_2a_ (#M80545), and Ca_V_β_4_ (#L02315) constructs were provided by Edward Perez-Reyes (University of Virginia). The D_4.4_R (#AF1199329) construct was provided by Hubert H. M. Van Tol (University of Toronto). D_2_R cDNA (#NM_000795) was obtained from the UMR cDNA Resource Center (https://www.cdna.org). Per well, a total of 0.5 μg of DNA (of which GFP cDNA was less than 10%) was used following the methods of Roberts-Crowley and Rittenhouse (2009) [13].

#### Electrophysiology

Whole-cell currents were recorded following the methods of Liu et al. [11]. High resistance seals were established in Mg^2+^ Tyrode’s (in mM): 5 MgCl_2_, 145 NaCl, 5.4 KCl, and 10 HEPES, brought to pH 7.50 with NaOH. Once a seal was established and the membrane ruptured, the Tyrode’s solution was exchanged for external bath solution (in mM): 125 NMG-aspartate, 20 Ba-acetate, 10 HEPES, brought to pH 7.50 with CsOH. Only cells with ≥ 0.2 nA of current were used. Data were acquired using Signal 2.14 software (CED) and stored for later analysis on a personal computer. Linear leak and capacitive currents were subtracted from all traces.

#### Drugs

All chemicals were purchased from Sigma unless otherwise noted. FPL 64176 (FPL), nimodipine (NIM), and oleoyloxyethyl phosphorylcholine (OPC, Calbiochem) were prepared as stock solutions in 100% ethanol. Quinpirole (quin) and Oxotremorine-M (Oxo-M, Tocris) were dissolved in DDW and stored as 10 mM stock solutions at − 70 °C. Stocks were diluted daily to the final concentration by at least 1000-fold with external solution. For ethanol-prepared stocks, the final ethanol concentration was less than 0.1%.

#### Statistical analysis

Data are presented as the mean ± s.e.m. Data were analyzed for significance using a Student’s paired *t*-test for two means, or a one-way ANOVA followed by a Tukey multiple-comparison post hoc test. Statistical significance was set at *p *< 0.05 or < 0.001. Analysis programs included Signal (CED), Excel (Microsoft), and Origin (OriginLab).

### Results

#### Characterization of recombinant Ca_V_1.3 current as L-type in HEK-M1 cells

Whole-cell L-currents, from β_3_-containing L-channels, elicited from a holding potential of − 60 mV to a test potential of − 10 mV, averaged − 4699 ± 279 pA (n = 3) compared to − 9 ± 1 pA for HEK-M1 cells transfected with only accessory subunits (n = 10, *P *< 0.001). Lack of current from cells transfected without Ca_V_1.3b, confirmed that HEK-M1 cells exhibit little endogenous Ca^2+^ current and transfection of accessory subunits does not upregulate endogenous Ca^2+^ channels. Recombinant current was confirmed as L-type by showing sensitivity to the L-VGCC antagonist NIM. NIM inhibited β_3_-containing currents (Additional file 1A) in a concentration-dependent manner (Additional file 1B). Currents were also sensitive to FPL, which enhanced current from β_2a_- and β_3_-containing channels and produced long-lasting tail currents upon repolarization (Additional file 1C, D). Additionally, FPL produced a slight hyperpolarizing voltage shift in the peak inward current and enhanced current amplitude at all voltages (Additional file 1E). Additional file 1F demonstrates that FPL enhanced the long-lasting tail current in a concentration-dependent manner. These pharmacological and biophysical properties show that transfection of HEK-M1 cells with Ca_V_1.3b and accessory subunits produce currents with L-type characteristics.

#### The Ca_V_β-subunit varies the magnitude of Ca_V_1.3 current inhibition by M_1_Rs

**Fig. 1:**
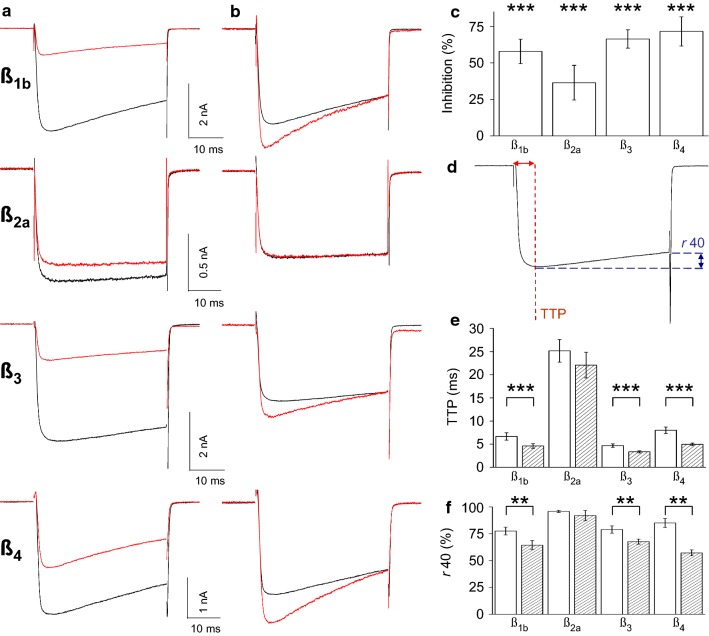
Ca_V_1.3b current inhibition and kinetic changes produced by M_1_R stimulation are Ca_V_β-subunit dependent. **a** Representative current traces from Ca_V_1.3b coexpressed with β_1b_, β_2a_, β_3_ or β_4_ before (black) or 1 min after applying 10 μM Oxo-M (red). **b** Current traces from **a** were normalized to the end of the test pulse. **c** Summary of Oxo-M inhibition of Ca_V_1.3b with different Ca_V_β-subunits. Maximal inward current amplitudes were measured after the onset of the test pulse using a trough seeking function (peak current). Percent of current inhibition was calculated as: *%I*_*inhib*_= 100*(I_*CTL*_–*I*_*DRUG*_*)∕I*_*CTL*_ , where I_CTL_ and I_DRUG_ are the average maximum current amplitude of 5 traces prior to and after 1 min of application of test material (unless otherwise noted). **d** Schematic of quantification of kinetic changes. **e**, **f** Summary of kinetic changes (n = 4–6, ***P < 0.001, **P < 0.05) open bars, control; hatched bars, Oxo-M. **e** Time to peak (TTP) was measured using a minimum seeking function in Signal within the test pulse duration. **f** Current remaining (*r*40) was measured from an average of five normalized current traces per condition using the equation: *r40 *= 100*I_*end*_*∕I*_*peak*_ , where *r*40 is the percent of the maximum inward current remaining at the end of a 40 ms test pulse; I_end_ is the current amplitude at the end of the test pulse; I_peak_ is the maximum inward current measured during the test pulse

In MSNs, M_1_R stimulation inhibits L-current in Ca_V_1.2 knockout animals [4]. Only Ca_V_1.2 and Ca_V_1.3 constitute the L-type Ca_V_α_1_ subunits expressed in brain [23], implying that M_1_Rs specifically inhibit Ca_V_1.3 current. Using a cell line transfected with only Ca_V_1.3 channels provides molecular proof for the identity of the inhibited channel. Therefore, to determine if activation of M_1_Rs inhibits Ca_V_1.3 activity, peak current amplitudes were measured prior to and following application of the M_1_R agonist Oxo-M. Figure 1a compares representative current traces for Ca_V_1.3b coexpressed with β_1b_, β_2a_, β_3_, or β_4_-subunits in the absence or presence of Oxo-M. After 1 min, Oxo-M significantly inhibited L-current by 58 ± 8% with β_1b_; 36 ± 12% with β_2a_; 66 ± 6% with β_3_; and 72 ± 10% with β_4_ (Fig. 1c). Oxo-M elicited kinetic changes that were visualized by normalizing individual traces to the end of the 40 ms test pulse (Fig. 1b), which were quantified by measuring TTP and *r*40 (Fig. 1d). TTP (Fig. 1e) and *r*40 (Fig. 1f) decreased following Oxo-M with β_1b_, β_3_, or β_4_; however, no changes were detected with β_2a_ (*P *≥ 0.11 for TTP; *P *≥ 0.40 for *r*40). These differences in the magnitude of current inhibition and kinetics suggest that the Ca_V_β-subunit affects M_1_R modulation of Ca_V_1.3b.

#### Dopamine D_2_ receptors inhibit Ca_V_2.2 but not Ca_V_1.3 currents

**Fig. 2:**
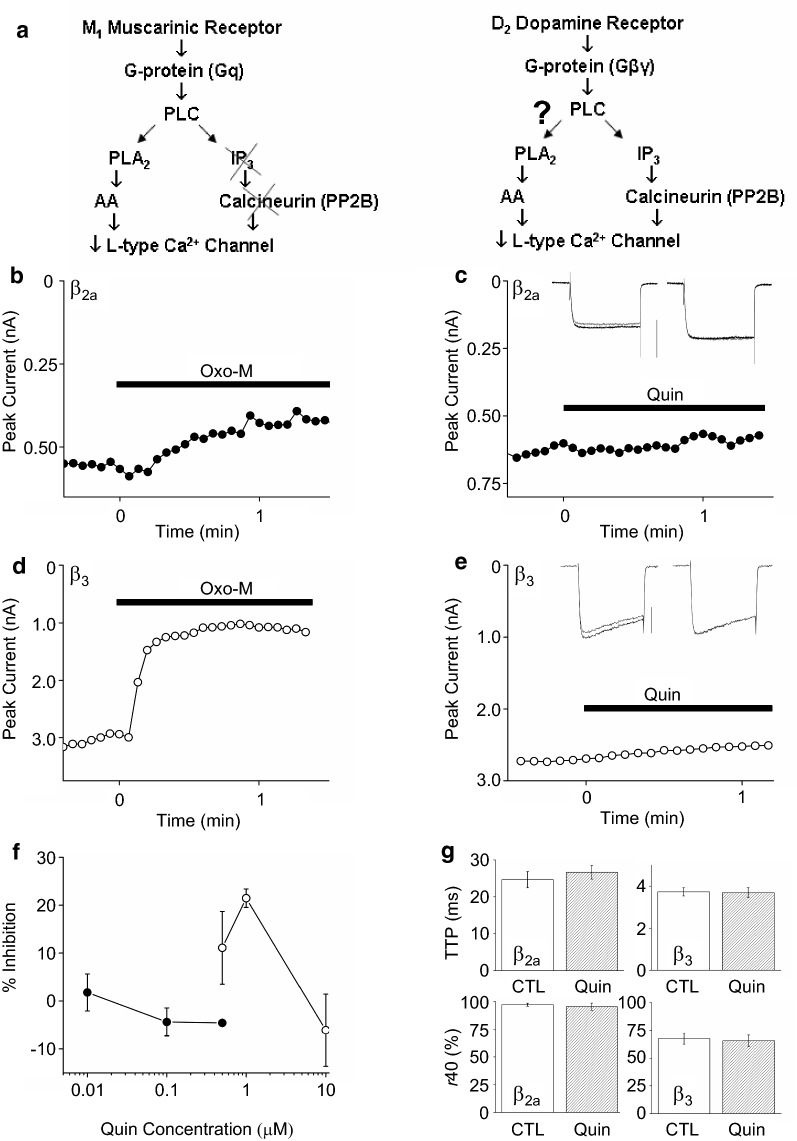
M_1_Rs but not D_2_Rs inhibit recombinant L-current. **a** Comparison of M_1_R and D_2_R signaling pathways that inhibit L-VGCC activity. **b** Time course of Oxo-M applied at time 0 for Ca_V_1.3b-β_2a_ current. **c** Time course of 10 nM quin applied at time 0 for Ca_V_1.3b-β_2a_ current. Inset: (*left*) Individual current traces before (black) and after 1 min of quin, scale bar = 0.5 nA. (*right*) Normalized traces. **d** Time course of Oxo-M applied at time 0 for Ca_V_1.3b-β_3_ current. **e** Time course of 0.5 μM quin applied at time 0 for Ca_V_1.3b-β_3_ current. Inset: same as **c**, scale bar = 1 nA. **f** Concentration–response curve of quin on Ca_V_1.3b-β_2a_ (filled circles) and Ca_V_1.3b-β_3_ (open circles) currents (n = 2–5). **g** Summary of kinetic analysis

Both M_1_Rs and D_2_Rs activate pathways involving G proteins, PLC, and AA release (Fig. 2a). However, whether L-current inhibition by D_2_Rs shows varied inhibition depending on Ca_V_β-subunit expression has not been examined. Therefore, we coexpressed D_2_Rs with Ca_V_1.3b, α_2_δ-1 and different Ca_V_β-subunits. While Oxo-M inhibited Ca_V_1.3b-β_2a_ currents over time (Fig. 2b), quin, a D_2_R agonist, had no effect on current amplitude (Fig. 2c) or kinetics (Fig. 2c inset, g). Since Ca_V_1.3b-β_2a_ current shows less inhibition and no kinetic changes with Oxo-M, we tested whether Ca_V_1.3b-β_3_ current was sensitive to modulation by quin. Figure 2d shows a time course of Ca_V_1.3b-β_3_ current inhibition by Oxo-M whereas the time course with quin (Fig. 2e) shows no inhibition or kinetic change (Fig. 2e inset, g). Several concentrations of quin were tested but did not inhibit L-current to the same extent as Oxo-M (Fig. 2f). D_2_Rs appeared to desensitize with 10 μM quin. Application of quin for 1 min to cells co-transfected with the D_2_R-like family member, D_4.4_R, inhibited L-current by 8.5 ± 2.5% and did not produce changes in TTP or *r*40 (Additional file 2).

**Fig. 3:**
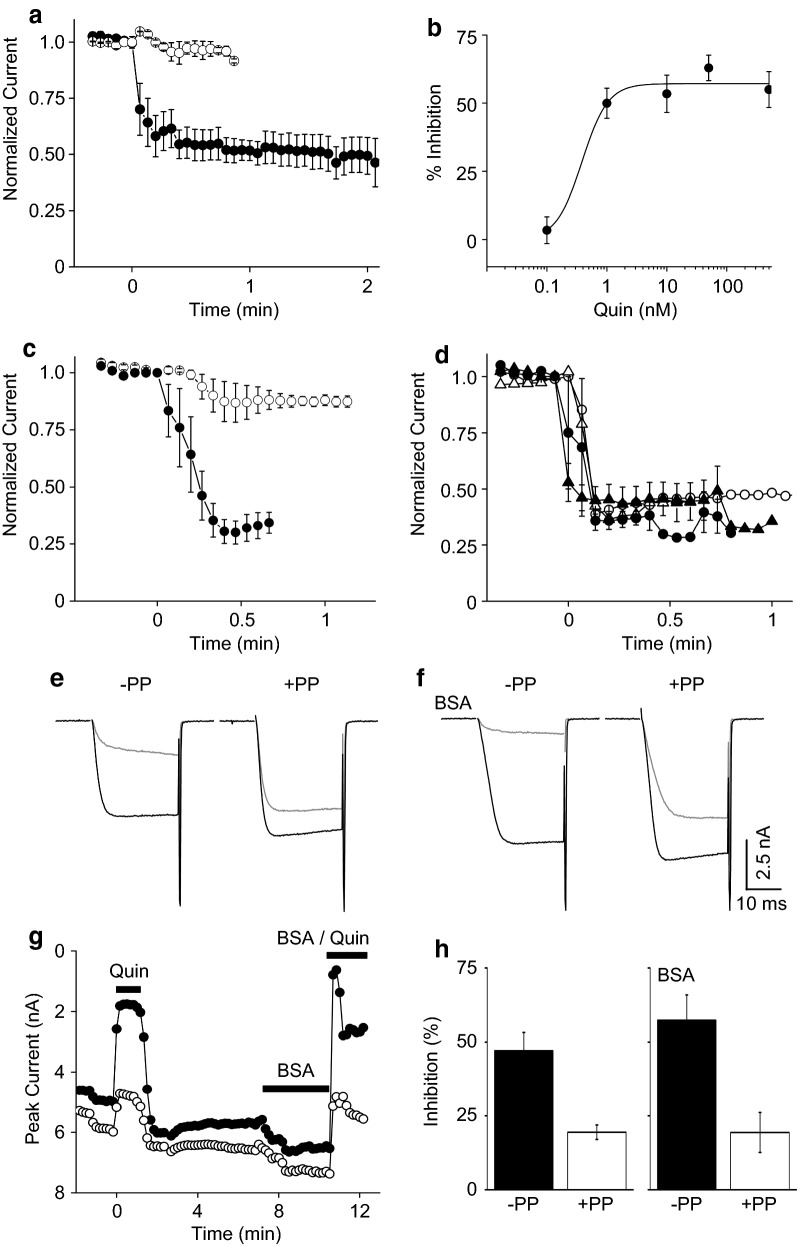
D_2_Rs and M_1_Rs inhibit recombinant N-current demonstrating successful expression of both GPCRs. To demonstrate that D_2_Rs are functional, HEK-M1 cells were transfected with the D_2_R, Ca_V_2.2, α_2_δ_1_, and a Ca_V_β subunit plasmids using the same conditions as described in the Methods section and in Additional file 1 legend as described for Ca_V_1.3b. **a** Time course of Ca_V_2.2-β_3_ current inhibition by 0.5 μM quin added at time 0 with (filled circles, n = 8, *P *< 0.001 compared to CTL) or without (open circles, n = 3) co-transfection of D_2_Rs. **b** Concentration–response curve of quin on Ca_V_2.2-β_3_ current (n = 3–5). **c** Time course of Ca_V_2.2-β_3_ current inhibition by Oxo-M added at time 0 under CTL conditions (filled circles, n = 5, *P *< 0.001 compared to CTL) or preincubation for at least 3 min with 10 μM of the PLA_2_ antagonist, OPC (open circles, n = 5, *P *< 0.05 compared to Oxo-M alone, ANOVA). **d** Time course of Ca_V_2.2-β_3_ current inhibition by 10 (filled triangles) or 50 nM (filled circles) quin under control conditions or preincubated with OPC (open symbols) (n = 1–5). **e** Representative Ca_V_2.2-β_2a_ currents measured at a test potential of + 20 mV (−PP) from a holding potential of − 90 mV. A 25 ms prepulse to + 120 mV was placed before a second test pulse (+PP) to measure for membrane-delimited inhibition. CTL current (black) or 30 s after application of 0.5 μM quin (grey). **f** Same as **e** in the presence of 1 mg/ml BSA. **g** Time course of Ca_V_2.2-β_2a_ current (−PP, filled circles; +PP, open circles) exposed to 0.5 μM quin at time 0 for 1 min. After washing, current fully recovered; BSA was added for 3 min before addition of BSA/quin. **h** Summary of Ca_V_2.2-β_2a_ inhibition by quin (n = 9) or BSA/quin (n = 3)

To confirm that lack of L-current inhibition was not due to poor expression of D_2_Rs, we repeated the experiment but substituted Ca_V_2.2 for Ca_V_1.3b to serve as a positive control since activated D_2_Rs also inhibit Ca_V_2.2 [24–26]. Quin inhibited Ca_V_2.2 by 45 ± 7% after 30 s and 48 ± 4% after 1 min (Fig. 3a). Inhibition occurred specifically by activating transfected D_2_Rs because cells transfected without D_2_Rs showed no response to quin (Fig. 3a, n = 3). Moreover, N-current inhibition by quin occurred in a concentration-dependent manner (Fig. 3b, n = 3–5). Compared to lower concentrations, 10 μM quin resulted in less inhibition; inhibited current did not recover upon wash, suggesting this concentration causes receptor desensitization (data not shown). Thus, our findings indicate that transfected D_2_Rs functionally express in HEK-M1 cells to modulate Ca_V_2.2, but not Ca_V_1.3b VGCC activity.

#### M_1_R and D_2_R pathways use different signaling mechanisms to inhibit N-current

To compare D_2_Rs and M_1_Rs signaling pathways on Ca_V_2.2 current, we first confirmed that activation of the stably transfected M_1_Rs could suppress N-current. Indeed, Oxo-M inhibited currents from β_3_-containing channels by 70 ± 5% after 30 s (Fig. 3c). When incubated with the PLA_2_ antagonist OPC, cells showed less N-current inhibition by Oxo-M, 14 ± 8% inhibition after 30 s (Fig. 3c). In contrast, low concentrations of quin still suppressed N-current in the presence of OPC (Fig. 3d). Inhibition was relieved by pre-pulse facilitation (Fig. 3e, g, h) and occurred in the presence of BSA, which acts as a scavenger of free AA (Fig. 3f–h), suggesting that quin mediates membrane-delimited inhibition of N-current. These findings suggest that M_1_Rs and D_2_Rs do not share a common pathway leading to N-current inhibition.

### Discussion

Previously, the Ca_V_1.3b splice variant of L-VGCCs, found in MSNs, had not been specifically tested for modulation by GPCRs. Here, using HEK-M1 cells, we present the novel finding that M_1_R stimulation inhibits Ca_V_1.3b L-current with the accessory Ca_V_β-subunit determining the magnitude of inhibition. In contrast, stimulation of transfected D_2_Rs with quin does not recapitulate L-current inhibition observed in MSNs [3]. Pharmacological sensitivity to both FPL and NIM confirmed that Ca_V_1.3b expressed in HEK-M1 cells behaves similarly to other recombinant Ca_V_1.3 VGCCs [22, 27].

We also report that N-current modulation by the D_2_R short splice variant appears similar to membrane-delimited inhibition by the D_2_R long form [24]. In this form of modulation, when G proteins are activated, Gβγ directly binds to and inhibits Ca_V_2.2 which can be reversed by strong prepulses [10, 28]. Indeed, D_2_R-mediated inhibition of Ca_V_2.2 was independent of PLA_2_, whereas blockers of PLA_2_ abolished inhibition by M_1_Rs. Thus, the membrane-delimited pathway may be at least partially responsible for the inhibition of Ca_V_2.2 by D_2_Rs in MSNs [25].

In our experiments, the short splice variant of Ca_V_1.3 (Ca_V_1.3b) was unaffected by activation of D_2_Rs, expressed in HEK-293 cells, similar to a previous report on Ca_V_1.3a, which has a longer C-terminus [24]. Since neither D_2_R-long inhibited Ca_V_1.3a [24], nor D_2_R-short inhibited Ca_V_1.3b (Fig. 2f), one possibility is that another channel/receptor combination occurs in vivo; however, D_2_R-long and short equally couple to G_i_ proteins [29]. On the other hand, Ca_V_1.3a binds a scaffolding protein found in the postsynaptic density of synapses known as Shank [30]. In MSNs, Ca_V_1.3a requires an association with Shank for current inhibition by D_2_Rs [4]. Although lack of the longer Ca_V_1.3 C-terminus may explain the absence of channel modulation by D_2_Rs in our studies, we found that Oxo-M inhibits Ca_V_1.3b currents, showing that this short splice variant of Ca_V_1.3 can be modulated by a G_q_PCR. Therefore, a missing intermediary protein vital for D_2_R modulation of Ca_V_1.3b may underlie the lack of inhibition reported here, or D_2_Rs may not modulate Ca_V_1.3b.

### Conclusions

These findings highlight the specific regulation of Ca^2+^ channels in a Ca_V_β-subunit dependent manner by different neurotransmitters. While M_1_R and D_2_R pathways contain similar signaling molecules and share a common functional output of inhibiting Ca^2+^ channels, differences between the two cascades exist. Expression and localization of Ca_V_1.3b associated with different Ca_V_β-subunits in a tissue or cell may dictate how Ca^2+^ influx is modulated by nearby GPCRs, ultimately affecting Ca^2+^-dependent processes.

## Limitations

Further experiments are needed to determine the differences in signaling between successful Ca_V_1.3b inhibition by M_1_Rs versus none with D_2_Rs.

## Additional files

**Additional file 1.** Pharmacological characterization of Ca_V_1.3b L-current. HEK-M1 cells were washed with DMEM and the DNA mixture of Ca_V_1.3b, α_2_δ-1, a β_3_-subunit and GFP was added and incubated for 1 h at 37 °C in a 5% CO_2_ incubator. Supplemented media, without antibiotics, was then returned to the cells to bring the volume up to 1 ml (normal medium volume). After 2 h, cells were washed with supplemented media and washed a final time 2 h later. 10 mM MgSO_4_ was added to the medium to block basal activity of Ca_V_1.3b, which helped minimize excitotoxicity of transfected cells. Cells were transferred 24–72 h post-transfection using 2 mM EDTA in 1X PBS, to poly-*l*-lysine-coated coverslips. Recording began 1 h after transfer to coverslips. **A** Individual traces of Ca_V_1.3b-β_3_ current before (CTL) and after exposure to 0.3 µM NIM. **B** Concentration–response curve of L-current inhibition to NIM (n = 4–8). **C** Ca_V_1.3b-β_2a_ currents before and after exposure to FPL (1 µM). Cells were stepped to a test potential of − 10 mV from a holding potential of − 90 mV followed by repolarization to − 90 or − 50 mV. Control (CTL) currents from β_2a_-containing L-VGCCs show little to no inactivation as observed previously [31]. **D** Ca_V_1.3b-β_3_ currents before and after FPL. Cells were stepped to a test potential of − 10 mV from a holding potential of − 60 mV followed by repolarization to − 60 mV. Following FPL, both β_2a_- and β_3_-containing channels exhibited slower activation and deactivation kinetics, hallmarks of agonist action on L-current [32]. **E** FPL enhancement of the Ca_V_1.3b-β_2a_ current–voltage plot from a holding potential of − 90 mV (CTL, filled circles; FPL, open circles, n = 3, *P < 0.05). **F** Concentration–response curve of Ca_V_1.3b-β_3_ tail current enhancement to FPL (n = 4–8). Currents inhibited by NIM and enhanced by FPL fully recovered by washing with bath solution (data not shown).

**Additional file 2.** D_4.4_Rs do not inhibit recombinant L-current. **A** Summary bar graph of Ca_V_1.3b-β_3_ current inhibition by 0.5 μM quin (n = 5). **B & C** Summary bar graphs of TTP and *r*40 kinetic analysis.

## Abbreviations

AA: arachidonic acid
cPLA_2_: Ca^2+^ dependent, cytosolic phospholipase A_2_
D_2_Rs: dopaminergic D_2_ receptors
FPL: FPL 64176
GFP: green fluorescent protein
GPCRs: G protein-coupled receptors
HEK-293 cells: human embryonic kidney cells
M_1_Rs: muscarinic M_1_ receptors
MSN: medium spiny neurons
NIM: nimodipine
NMG: N-methyl-*D*-glucamine
OPC: oleoyloxyethyl phosphorylcholine
Oxo-M: Oxotremorine-M
PLA_2_: phospholipase A_2_
PLC: phospholipase C
PTX: pertussis toxin
Quin: quinpirole
SCG: superior cervical ganglion
TTP: time to peak
VGCCs: voltage-gated Ca^2+^ channels
