# Supplementary figures and images for: Modulation of CaV1.3b L-type calcium channels by M1 muscarinic receptors varies with CaVβ subunit expression
Source: BMC Res Notes. 2018 Sep 27;11:681. doi: 10.1186/s13104-018-3783-x (PMC6161362; doi:10.1186/s13104-018-3783-x)

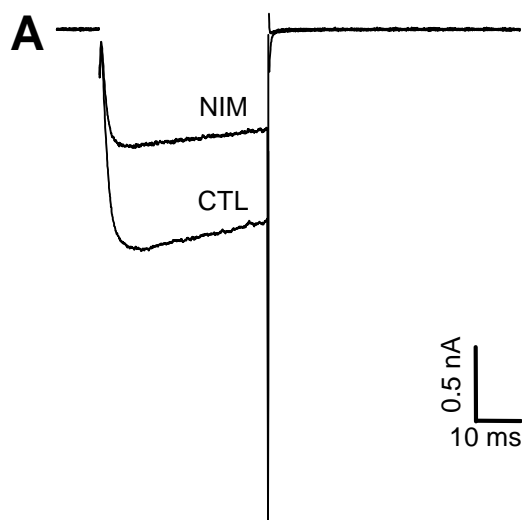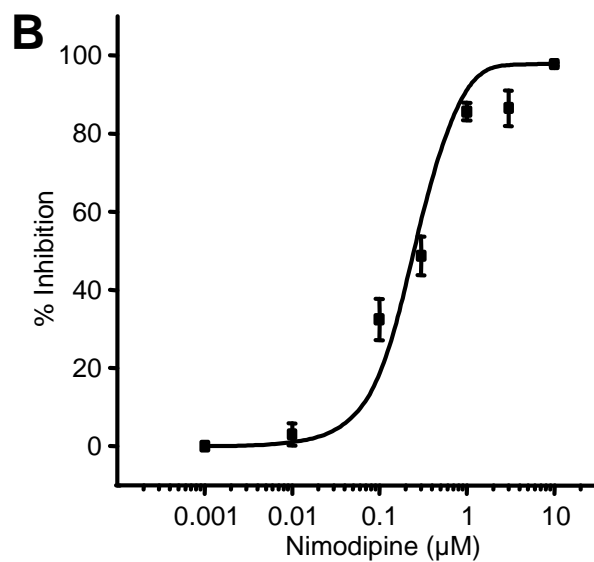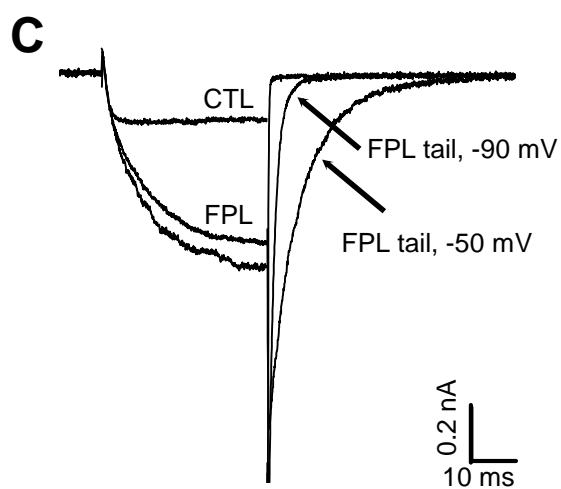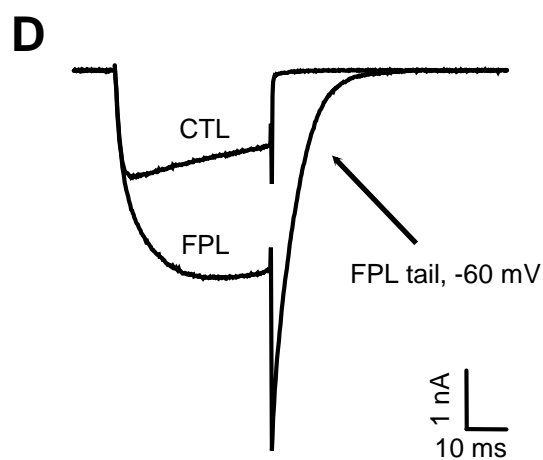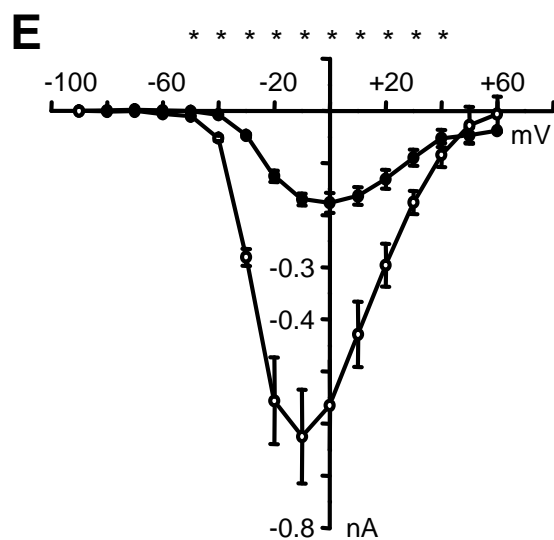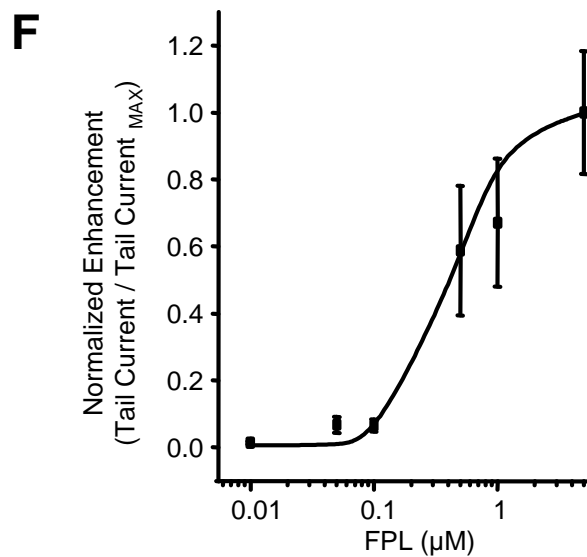

Supplement: Supplementary file 1 — Additional file 1. Pharmacological characterization of CaV1.3b L-current. HEK-M1 cells were washed with DMEM and the DNA mixture of CaV1.3b, α2δ-1, a β3-subunit and GFP was added and incubated for 1 h at 37 °C in a 5% CO2 incubator. Supplemented media, without antibiotics, was then returned to the cells to bring the volume up to 1 ml (normal medium volume). After 2 h, cells were washed with supplemented media and washed a final time 2 h later. 10 mM MgSO4 was added to the medium to block basal activity of CaV1.3b, which helped minimize excitotoxicity of transfected cells. Cells were transferred 24–72 h post-transfection using 2 mM EDTA in 1X PBS, to poly-l-lysine-coated coverslips. Recording began 1 h after transfer to coverslips. A Individual traces of CaV1.3b-β3 current before (CTL) and after exposure to 0.3 µM NIM. B Concentration–response curve of L-current inhibition to NIM (n = 4–8). C CaV1.3b-β2a currents before and after exposure to FPL (1 µM). Cells were stepped to a test potential of − 10 mV from a holding potential of − 90 mV followed by repolarization to − 90 or − 50 mV. Control (CTL) currents from β2a-containing L-VGCCs show little to no inactivation as observed previously [31]. D CaV1.3b-β3 currents before and after FPL. Cells were stepped to a test potential of − 10 mV from a holding potential of − 60 mV followed by repolarization to − 60 mV. Following FPL, both β2a- and β3-containing channels exhibited slower activation and deactivation kinetics, hallmarks of agonist action on L-current [32]. E FPL enhancement of the CaV1.3b-β2a current–voltage plot from a holding potential of − 90 mV (CTL, filled circles; FPL, open circles, n = 3, *P < 0.05). F Concentration–response curve of CaV1.3b-β3 tail current enhancement to FPL (n = 4–8). Currents inhibited by NIM and enhanced by FPL fully recovered by washing with bath solution (data not shown). [file 13104_2018_3783_MOESM1_ESM.pdf]

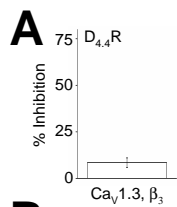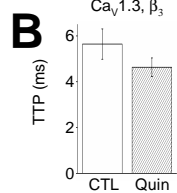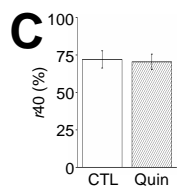

Supplement: Supplementary file 2 — Additional file 2. D4.4Rs do not inhibit recombinant L-current. A Summary bar graph of CaV1.3b-β3 current inhibition by 0.5 μM quin (n = 5). B & C Summary bar graphs of TTP and r40 kinetic analysis. [file 13104_2018_3783_MOESM2_ESM.pdf]
